# Dynamics and stationary configurations of heterogeneous foams

**DOI:** 10.1371/journal.pone.0215836

**Published:** 2019-04-29

**Authors:** Dong Wang, Andrej Cherkaev, Braxton Osting

**Affiliations:** Department of Mathematics, University of Utah, Salt Lake City, UT, United States of America; Delft University of Technology, NETHERLANDS

## Abstract

We consider the variational foam model, where the goal is to minimize the total surface area of a collection of bubbles subject to the constraint that the volume of each bubble is prescribed. We apply sharp interface methods to develop an efficient computational method for this problem. In addition to simulating time dynamics, we also report on stationary states of this flow for ≤ 21 bubbles in two dimensions and ≤ 17 bubbles in three dimensions. For small numbers of bubbles, we recover known analytical results, which we briefly discuss. In two dimensions, we also recover previous numerical results, computed using other methods. Particular attention is given to locally optimal foam configurations and heterogeneous foams, where the volumes of the bubbles are not equal. Configurational transitions are reported for the quasi-stationary flow where the volume of one of the bubbles is varied and, for each volume, the stationary state is computed. The results from these numerical experiments are described and accompanied by many figures and videos.

## Introduction

We consider the model for a *d*-dimensional foam (*d* = 2, 3) comprised of *n* bubbles, ${\left\{\Omega_{i}\right\}}_{i=1}^{n}$, each with a prescribed volume, $H^{d}\left(\Omega_{i}\right)=V_{i}$, that arrange themselves as to minimize the total surface area,

$$\begin{array}{c} \min_{H^{d}\left(\Omega_{i}\right)=V_{i}}\;H^{d-1}\left(\cup_{i=1}^{n}\partial \Omega_{i}\right). \end{array}$$ (1)

Here we have denoted the *d*-dimensional Hausdorff measure by $H^{d}$. Note that in (1), the interfaces between bubbles and the interface between the bubbles, $\cup_{i=1}^{n}\partial \Omega_{i}$ and the rest of Euclidean space, $R^{d}\backslash \cup_{i=1}^{n}\partial \Omega_{i}$, receive equal weight. We refer to stationary solutions of (1) as *stationary *n*-foams*. If the areas are all equal, we say the foam is *equal-area* and otherwise we say the foam is *heterogeneous*. The isoperimetric variational problem (1) is classical; its history and the state of known results can be found in the recent book [1]. The two-dimensional problem is discussed in [2–4], the three-dimensional problem is discussed in [5, 6], and the higher-dimensional *n* = 2 problem is discussed in [7]. We’ll further review the most relevant of these results in the Background Section.

In this paper, we apply sharp interface methods from computational geometry to investigate (1); these methods are described in the Computational Methods Section. In particular, we study an approximate gradient flow of (1) in dimensions *d* = 2, 3, which gives the time-evolution of a foam for a given initial configuration. This corresponds to a volume-constrained mean curvature flow of the interfaces between bubbles. Examples of such time-dynamics for equal-area, two- and three- dimensional foams are given for *n* = 12 and *n* = 8 respectively.

We also study stationary foams of the gradient flow. In two dimensions, we recover many of the results from [3], where candidate solutions for the equal-area problem (1) for many values of *n* were found using very different computational methods then the present work. Stationary configurations of two-dimensional, equal-area *n*-foams for *n* = 2, …, 21 are given. Particular emphasis is given to the existence of multiple stationary foams that correspond to geometrically distinct configurations but have similar total surface areas. For example, a second two-dimensional, *n* = 16-foam with slightly larger total perimeter is presented. Our computational methods also extend to three dimensions; stationary foams for the equal-area problem for *n* = 1, …, 17 are computed. As far as we know, these results are new for *n* ≥ 5.

To further study multiple stationary foams, we consider heterogeneous foams. In particular, we study the *quasi-stationary flow* where the area of one of the bubbles is slowly varied and for each area, the stationary solution is computed. We observe *configurational transitions* where there are sudden changes in the stationary foams in this quasi-stationary flow. A comparison of two different quasi-stationary flows between an *n* = 6 and *n* = 7 equal-area foam is presented.

## Background

In this section, we review some relevant previous results in two and three dimensions.

### Two dimensional results

In 1993, Foisy, Alfaro, Brock, Hodges, and Zimba proved that the equal-area 2-foam in two dimensions is given by two intersecting discs separated by a line so that all angles are 120° [2]. In 2004, Wichiramala showed that the equal-area 3-foam in two dimensions is given by three intersecting discs so that all angles are 120° [4]. For a two-dimensional *n*-foam with *n* ≥ 4, the optimal domain isn’t known analytically, but, for small values of *n*, candidate solutions have been computed numerically [3].

For all *n*, necessary conditions for any minimizer are given by Plateau’s laws:

(i) each interface between bubbles has constant curvature and
(ii) interfaces between bubbles meet in threes at vertices with equal angles.

We give a brief and formal derivation of Plateau’s laws here; our goal is to give an accessible discussion that we can refer to when analyzing the numerical results.

Given *n* bubbles, Ω_1_, …, Ω_*n*_, with given areas *V*_1_, …, *V*_*n*_, *i.e*., $\int_{\Omega_{i}}dx=V_{i}$, our variational problem is to find the configuration that has minimal total length of the interface Γ that separates the bubbles. We assume that the bubbles are all contained in a region Ω, and denote the complement of the bubbles in Ω by $\Omega_{0}=\Omega \backslash \cup_{i=1}^{n}\Omega_{i}$. The interface Γ is the union of the shared boundaries Γ_*i*,*j*_ between all neighboring domains Ω_*i*_ and Ω_*j*_ as well as the outer interfaces Γ_*i*0_ of the external domains Ω_*i*_ with Ω_0_. The total length of the boundary is $J\left(\Omega_{i}\right)=\sum_{\begin{array}{c} i,j=0\\ i\neq j \end{array}}^{n}\int_{\Gamma_{i,j}}ds$. The variational problem can then be written

$$\left\{ \begin{array}{cc} \min_{\Omega_{i}} & \;J(\Omega_{i})\\ \text{subject}\;\text{to} & \;\int_{\Omega_{i}}dx=V_{i},\;i=1,\ldots ,n. \end{array}\right.$$ (2)

### Interfaces have constant curvature

Introducing the Lagrange multipliers λ_*i*_, we formulate the Lagrangian for (2),

$$L(\Omega_{i})=J\left(\Omega_{i}\right)+\sum_{i=1}^{n}\lambda_{i}(\int_{\Omega_{i}}dx-V_{i})$$ (3)

To see how the Lagrangian in (3) changes as we vary the domains Ω_*i*_, we first recall the formulas for the shape derivative of the area and perimeter with respect to changes in the domain. Consider a domain Ω with piecewise smooth boundary Γ and let *s* be the distance along the boundary. We consider the infinitesimal deformation of the domain in the direction of a velocity field *V*, which moves a point *x* on the boundary Γ to the point $x+\varepsilon (V\left(x\right)\cdot \hat{n}\left(x\right))\hat{n}\left(x\right)$, where *ε* > 0 is small and $\hat{n}$ is the normal vector to Γ. In other words, the point *x* on the boundary of Γ is moving in the normal direction at speed *εc*(*x*) where $c\left(x\right)=V\left(x\right)\cdot \hat{n}\left(x\right)$. The resulting change in the area of Ω, *δ*|Ω|, and the change in the arc length of Γ, *δ*|Γ|, are given by

$$\begin{array}{c} \delta |\Omega |=\varepsilon \int_{\Gamma }c\left(x\right)\;ds+o\left(\varepsilon \right),\;\text{and}\;\delta \left|\Gamma \right|=\varepsilon \int_{\Gamma }\kappa \left(s\right)c\left(x\right)\;ds+o\left(\varepsilon \right), \end{array}$$

where *κ*(*s*) denotes the curvature of Γ.

Using these shape derivatives, and looking for critical points of the Lagrangian *L* in (3) due to a variation of the boundary Γ_*i*,*j*_ between Ω_*i*_ and Ω_*j*_, we arrive at the condition

$$\begin{array}{c} \int_{\Gamma_{i,j}}\left(\lambda_{i}-\lambda_{j}+\kappa_{i,j}\right)c\left(s\right)\;ds=0,\;\;\forall i,j=1,\ldots ,n \end{array}$$

where *κ*_*i*,*j*_ is the curvature of the boundary between domains *i* and *j* and *c*(*s*) is the speed of variation at the point *s* on an interface Γ_*i*,*j*_. Since this condition should hold for all *c*(*s*), we arrive at the optimality condition

$$\begin{array}{c} \kappa_{i,j}=\lambda_{i}-\lambda_{j}=\text{constant} \end{array}$$ (4)

The optimality condition (4) implies that (i) the outer interfaces Γ_*i*0_ of the external domains Ω_*i*_ with Ω_0_ are arcs of circles, (ii) the shared boundaries Γ_*i*,*j*_ between all neighboring domains Ω_*i*_ and Ω_*j*_ are arcs of circles, and, in particular, (iii) the interfaces between congruent bubbles are straight lines. The value of the Lagrange multiplier, λ_*i*_, depends on the size of the domain Ω_*i*_ as well as on its position in the foam. In particular, the interface between a larger and smaller bubble should “bend towards” the larger shape. We have that $\lambda_{i}\to \{\begin{array}{cc} 0 & |\Omega_{i}|\to \infty \\ \infty & |\Omega_{i}|\to 0 \end{array}$.

### Triple junctions have equal angles

Finding optimal angles between the arcs of three domains that meet at a single point requires a separate variational argument, analogous to the Weierstrass test [8]. Assume that three boundary arcs Γ_1_, Γ_2_, and Γ_3_ meet at a point *z* and consider a ball *B*_*ε*_ of radius *ε* centered at *z*. We now fix the ball *B*_*ε*_ and the three points *x*_*i*_ = Γ_*i*_ ∩ ∂*B*_*ε*_ for *i* = 1, 2, 3. We will minimize the Lagrangian, *L*, in (3) by varying the position of *z* ∈ *B*_*ε*_. The change of the areas of the domains Ω_*i*_ is *O*(*ε*^2^) while the variation of the boundary lengths is *O*(*ε*); therefore the contribution of the increment of areas within *B*_*ε*_ can be neglected. Next, the variation of the interface lengths are approximated (up to *o*(*ε*)) by the variation of distances |*x*_*i*_ − *z*|. We arrive at the local problem:

$$\begin{array}{c} \min_{z}\;j\left(z\right),\;\text{where}\;j\left(z\right)=\sum_{i=1}^{3}\left|x_{i}-z\right|. \end{array}$$

First, we observe that sum of any two angles between Γ_1_, Γ_2_ and Γ_3_ is smaller than 180°. If an angle is larger than 180°, than all three circumferential points *x*_1_ and *x*_2_ and *x*_3_ lie on one side of the ball in a half-disc. Such a configuration cannot be optimal because all three lengths can be decreased by simply shifting the point *z* towards the middle point, *x*_2_. If the angles are such that any two of them are smaller that 180°, the optimal intersection point *z* is in the ball *B* and may be found from the condition

$$\begin{array}{c} \nabla j\left(z\right)=0\;\Rightarrow \;\sum_{i=1}^{3}\frac{x_{i}-z}{|x_{i}-z|}=0. \end{array}$$

That is, the sum of the three unit vectors is zero, which implies that the angle between any two of them is 120°. One can also show that in a stationary foam, four or more bubbles cannot meet at a single point.

*Remark* 0.1. The honeycomb structure satisfies the necessary conditions for optimality and is the optimal configuration of equal-area bubbles as *n* → ∞ [9].

### Three dimensional results

In three dimensions, less is known about optimal foam configurations. The double bubble conjecture was proven in 2002 by M. Hutchings, F. Morgan, M. Ritore, and A. Ros [6]. The necessary conditions for any minimizer are referred to as Plateau’s laws:

(i) interfaces between bubbles have constant mean curvature,
(ii) bubbles can meet in threes at 120° angles along smooth curves, called Plateau borders, and
(iii) bubbles can meet in fours and the four corresponding Plateau borders meet pairwise at angles of cos^−1^(−1/3) ≈ 109°.

In what follows, we give a brief and formal derivation of these conditions here; a rigorous proof was given by Taylor [5].

As in the two-dimensional case, we consider *n* bubbles Ω_1_, … Ω_*n*_, with given volumes *V*_1_, …, *V*_*n*_, *i.e*., $\int_{\Omega_{i}}dx=V_{i}$. Our goal is to find the configuration that has minimal total surface area of the interfaces, Γ = ∪Γ_*i*,*j*_, that separate the bubbles. Again, the interfaces consists of the shared components Γ_*i*,*j*_ of two neighboring domains Ω_*i*_ and Ω_*j*_ for *i*, *j* = 1, …, *n* and the interfaces Γ_*i*,0_ of an external bubble Ω_*i*_ with the complement, Ω_0_. Introducing a multiplier λ_*i*_ for each volume constraint, the Lagrangian for this variational problem is given by

$$\begin{array}{c} L\left(\Omega_{i}\right)=\sum_{\begin{array}{c} i,j=0\\ i\neq j \end{array}}^{n}\;\;\int_{\Gamma_{i,j}}ds+\sum_{i=1}^{n}\lambda_{i}(\int_{\Omega_{i}}dx-V_{i}), \end{array}$$ (5)

where *ds* is an element of the interface Γ_*i*,*j*_.

### Interfaces have constant mean curvature

Taking the shape derivative of the Lagrangian in (5) and looking for critical points, an similar argument to the one given for two dimensions yields the stationary conditions

$$\begin{array}{c} \kappa_{i,j}=\lambda_{i}-\lambda_{j}\;\text{on}\;\Gamma_{i,j}. \end{array}$$

Here *κ*_*i*,*j*_ is the mean curvature of the interface of Γ_*i*,*j*_ (compare with (4)). This condition states that the mean curvature of each interface, Γ_*i*,*j*_, is constant.

*Remark* 0.2. Minimal surfaces are a special case of the problem under study. Here, the constraints on volumes are not imposed; therefore the minimal surface problem corresponds to λ_*i*_ = 0, ∀*i* and has the well-known optimality condition: *κ* = 0.

### Three bubbles meeting along a curve

We consider three smooth boundaries ∂Ω_*i*_, ∂Ω_*j*_, and ∂Ω_*k*_ intersecting along a curve *γ*, referred to as a *Plateau border*. The conditions of optimality at the Plateau border *γ* can be found from local variations inside an infinitesimal cylinder around the curve. The variation in an infinitesimal cylinder results in a change in the surface area that dominates the change in volume. Therefore, the necessary condition is identical to the corresponding well-studied condition for the minimal surface problem. At any point of the Plateau border *γ*, the sum of the three normal vectors ${\hat{n}}_{i}$ to the intersecting surfaces ∂Ω_*i*_ is zero and these vectors are orthogonal to the tangent $\hat{t}$ of *γ*:

$$\begin{array}{c} \sum_{i=1}^{3}{\hat{n}}_{i}=0,\;{\hat{n}}_{i}\cdot \hat{t}=0,\;i=1,2,3. \end{array}$$

This implies that ${\hat{n}}_{i}\cdot {\hat{n}}_{j}=-\frac{1}{2},\;\left(i\neq j\right)$ and the angle between the normals is 120°.

### Four bubbles meeting at a point

Similarly, we can consider a vertex where four bubbles intersect. Again, taking variations inside an infinitesimal ball around the vertex, we find that the sum of the four tangential vectors ${\hat{t}}_{i}$ to the Plateau borders is zero: $\sum_{i=1}^{4}{\hat{t}}_{i}=0.$ This condition implies that the tangential vectors are the directions from the center of a regular tetrahedron to its vertices. Thus, ${\hat{t}}_{i}\cdot {\hat{t}}_{j}=-\frac{1}{3},\;\left(i\neq j\right)$ and the angle *ϕ* between any two tangent vectors is $\phi =\arccos\left(-\frac{1}{3}\right)\approx 109^{{}^{\circ}}$.

*Remark* 0.3. Kelvin’s packing of truncated octahedra satisfy the necessary conditions for optimality [10]. The Weaire–Phelan structure also satisfies the necessary conditions for optimality and is the partition of three dimensional space with smallest known total surface area; it has 0.3% smaller total surface area than Kelvin’s structure [11].

## Computational methods

In this section, we discuss computational methods for the foam model problem (1). Here, the goal is to find interfaces between adjacent bubbles such that the total interfacial area is minimal with the constraint that the volume of each bubble is fixed. To design a numerical algorithm for (1), the first consideration is the method to represent the interfaces between bubbles. For contrast, we review several choices before describing the method used in the present work.

### Previous results

One method, known as the *front tracking method*, uses a discrete set of points to represent the interfaces [12]. Then, the energy is minimized by moving the points in the normal direction of the interface subject to some constraints. Although this idea is simple and straightforward, a number of difficult and complicated issues arise when dealing with multiple bubbles and possible topological changes, especially in three-dimensional simulations.

In [13], the author developed and implemented a method, referred to as the *Surface Evolver*, for solving a class of problems, including (1). A surface in this method is represented by the union of simplices and physical quantities (*e.g*., surface tension, crystalline integrands, and curvature) are computed using finite elements. The surface evolver iteratively moves the vertices using the gradient descent method, thus changing the surface. Although this idea is simple and straightforward, a number of difficult and complicated issues arise when dealing with multiple bubbles and possible topological changes.

Another approach is the *level set method*, where the interfaces is represented by the zero-level-set of a function *φ* [14]. This function is evolves in time according to a partial differential equation of Hamilton-Jacobi type,

$$\frac{\partial \varphi }{\partial t}=V_{n}\left|\nabla \varphi \right|.$$

Here, |⋅| is the Euclidean norm, ∇ denotes the spatial gradient, and *V*_*n*_ is the normal velocity. This type of method can easily handle topology changes because the interface is implicitly determined by the zero-level-set of the function *φ*. However, it is difficult to deal with the interface motion near multiple junctions and this type of method also needs to be reinitialized at each step or after every few steps.

Another option is to use the *phase field approach* where the interface is represented by a level-set of an order parameter function, *ϕ*; see, *e.g*., [15]. Here, *ϕ* takes two distinct values (*e.g*., ±1) for the two-phase case or several distinct vectors in the multiple-phase case. The function *ϕ* then evolves according to the Cahn-Hillard or Allen-Cahn equation, where a potential enforce that the function *ϕ* smoothly changes between the distinct values (or vectors) in a thin *ε*-neighborhood of the interface. This approach is simple and insensitive to topological changes. However, if the evolution of multiple junctions with arbitrary surface tensions needs to be resolved, it is difficult to find a suitable multi-well potential. Also, since it is desirable for *ε* to be small, a very fine mesh is needed to resolve the interfacial layer of width *ε*. Consequently, this algorithm is computationally expensive.

In [3], the authors iterated a shuffling-and-relaxation procedure to gradually find a candidate foam. At each iteration, they selected the shortest side and applied to it a neighbor-swapping topological process followed by relaxing the configuration in a quadratic mode. In the two-dimensional case, many nice candidates for various *n* are presented in [3]. However, this method requires a careful choice of both the initial configuration and the shuffling procedure is heuristic. The candidate configuration highly relied on the initial “circular” configuration. Also, it appears that this procedure needs a large number of iterations to reach a stationary candidate. It would be challenging to apply these ideas to the three-dimensional or heterogeneous foams.

### Computational method

In this paper, we use computational methods that are based on the *threshold dynamics methods* developed in [16–19]. Here, *n* indicator functions are used to denote the respective regions of each bubble in an *n*-foam. Additionally, we fix a rectangular box, $\Omega \subset R^{d}$ (*d* = 2, 3), which contains the supports of these *n* indicator functions and add an (*n* + 1)-th indicator function to denote the complement of the *n*-foam. Let *u* = (*u*_1_, *u*_2_, ⋯, *u*_*n*+1_) denote these indicator functions. We define

$$\begin{array}{c} B=\{u\in BV\left(\Omega \right):u_{i}\left(x\right)=\left\{0,1\right\},\;\sum_{i=1}^{n+1}u_{i}=1,\;a.e.\;x\in \Omega ,\;\text{and}\int_{\Omega }u_{i}\left(x\right)\;dx=V_{i},\;i\in \left[n+1\right]\}, \end{array}$$

where *V*_*i*_ is the prescribed volume of the *i*-th bubble for *i* ∈ [*n* + 1]. The constraints that *u*_*i*_(*x*) ∈ {0, 1} and ∑_*i*_ *u*_*i*_ = 1 together force the indicator functions to have disjoint support—which is equivalent to their representative domains being disjoint. We approximate the surface area of the interface between the *i*-th and *j*-th bubbles by $H^{d-1}\left(\partial \Omega_{i}\cap \partial \Omega_{j}\right)\approx L\left(u_{i},u_{j}\right)$, with

$$\begin{array}{c} L\left(u_{i},u_{j}\right)≔\frac{\sqrt{\pi }}{\sqrt{\tau }}\int_{\Omega }u_{i}\left(x\right)\left(G_{\tau }*u_{j}\right)\left(x\right)\;dx,\;\text{where}\;\;G_{\tau }\left(x\right)=\frac{1}{{\left(4\pi \tau \right)}^{\frac{d}{2}}}\exp(-\frac{{\left|x\right|}^{2}}{4\tau }). \end{array}$$ (6)

The Γ convergence of (6) to the interfacial area was proven in [19–21]. Using (6), the optimization problem (1) can be approximated as

$$\begin{array}{c} \min_{u\in B}\;E^{\tau }\left(u\right),\;\text{where}\;E^{\tau }\left(u\right)=\sum_{\begin{array}{c} i,j=0\\ i\neq j \end{array}}^{n+1}L\left(u_{i},u_{j}\right). \end{array}$$ (7)

Since the energy functional $E^{\tau }\left(u\right)$ is concave, we can relax the constraint set in (7) to obtain the equivalent problem [19, 22, 23],

$$\begin{array}{c} \min_{u\in K}\;E^{\tau }\left(u\right), \end{array}$$ (8)

where

$$K=\{u\in BV\left(\Omega \right):u_{i}\left(x\right)\in \left[0,1\right],\;\sum_{i}^{n+1}u_{i}=1,\;a.e.\;x\in \Omega ,\;\text{and}\;\int_{\Omega }u_{i}\left(x\right)\;dx=A_{i},i\in \left[n+1\right]\}$$

is the convex hull of B. The sequential linear programming approach to minimizing $E^{\tau }\left(u\right)$ is to consider a sequence of functions ${\left\{u^{s}≔\left(u_{1}^{s},u_{2}^{s},\ldots ,u_{n+1}^{s}\right)\right\}}_{s=0}^{\infty }$ which satisfies

$$\begin{array}{c} u^{s+1}=\arg\min_{u\in K}\;L_{u^{s}}\left(u\right) \end{array}$$ (9)

where $L_{u^{s}}^{\tau }\left(u\right)$ is the linearization of $E^{\tau }$. In this case,

$$L_{u^{s}}^{\tau }\left(u\right)=\sum_{i=1}^{n+1}\int_{\Omega }\Psi_{i}^{s}\left(x\right)u_{i}\left(x\right)\;dx,\;\text{where}\;\Psi_{i}^{s}=\sum_{\begin{array}{c} j=1\\ j\neq i \end{array}}^{n+1}G_{\tau }*u_{j}^{s}=G_{\tau }*(1-u_{i}^{s}).$$

Since *u*^*s*^ is given, (9) is a linear minimization problem. If we were to neglect the volume constraints, (9) could be solved point-wisely by setting

$$\begin{array}{c} u_{i}^{s+1}\left(x\right)=\{\begin{array}{cc} 1 & \text{if}\;\Psi_{i}^{s}\left(x\right)=\min_{k\in [n+1]}\Psi_{k}^{s}\left(x\right);\\ 0 & \text{otherwise}. \end{array} \end{array}$$ (10)

However, this solutions generally doesn’t satisfy the volume constraints.

Motivated by the schemes for the volume-preserving, two-phase flow [24–26], to find a solution $u^{s+1}\in B$ (*i.e*., each $u_{i}^{s+1}$ satisfies the corresponding volume constraint), Jacobs et. al. proposed an efficient auction dynamics scheme to impose the volume constraints for the multiphase problem [27]. In particular, they developed a membership auction scheme to find *n* + 1 constants λ_*i*_, *i* ∈ [*n* + 1] such that the solution $u^{s+1}\in B$ can be solved by

$$\begin{array}{c} u_{i}^{s+1}\left(x\right)=\{\begin{array}{cc} 1, & \text{if}\;\Psi_{i}^{s}\left(x\right)+\lambda_{i}=\min_{k\in [n+1]}\;\left(\Psi_{k}^{s}\left(x\right)+\lambda_{k}\right)\\ 0, & \text{otherwise}. \end{array} \end{array}$$ (11)

The algorithm is summarized in Algorithm 1 and we refer to [27] for details of the derivation.

The computational complexity of Algorithm 1 is *O*(*nM* log *M*), where *n* is the number of bubbles and *M* is the total number of grid points, which can be seen as follows. In the first step of Algorithm 1, we calculate *n* + 1 convolutions, each of which can be efficiently evaluated using the fast Fourier transform (FFT) with a total computational complexity of *O*((*n* + 1)*M* log *M*). In the second step, for each grid point *x*, we need to sort the value of Φ_*j*_(*x*) − λ_*j*_ several times, which can be accomplished using a quick-sort algorithm, with complexity *O*(*M*(*n* + 1)log(*n* + 1)). The complexity of the first step dominates.

Algorithm 1 was also proven to be unconditionally stable for any *τ* > 0 in [27]. In our implementation, we choose a value for *τ* as follows. On one hand, the algorithm can easily become “frozen” if *τ* is very small because, in the discretized space, *τ* is so small that no point can switch from one bubble to another (*i.e*., *u*_*i*_ changes from 0 to 1 or 1 to 0 for some *i*). On the other hand, for large *τ*, the interface easily moves but has large approximation error. In practice, we set *τ* ∼ *O*(*δx*) where *δx* is the mesh size.

**Algorithm 1**: Auction dynamics algorithm for solving (7) [27, Algorithm 1, 2].

**Input**: Let Ω_*n*_ be the discretization of the domain Ω, *n* be the number of grid points, $u^{0}=\left(u_{1}^{0},\ldots ,u_{n+1}^{0}\right)$ be the indicator functions for an initial *n* + 1-partition, *τ* > 0 be the time step, *V*_*i*_ for *i* ∈ [*n* + 1] be the prescribed volumes, *ε*_0_ be the initial value of *ε*, *α* be the *ε*-scaling factor, and *ε*_min_ be the auction error tolerance.

**Output**: $u^{S}\in B$ that minimizes (7).

Set *s* = 1

Set $\bar{\varepsilon }=\varepsilon_{\min}/n$

**while** *not converged* **do**

 **1. (Diffusion step)** Compute the coefficient functions,

$$\begin{array}{c} \Phi_{i}=1-\Psi_{i}^{s}=G_{\tau }*u_{i}^{s},\;\;i\in \left[n+1\right] \end{array}$$

 **2. (Find λ using auction dynamics)**

 Set λ_*i*_ = 0 for *i* ∈ [*n* + 1]

 Set *ε* = *ε*_0_

 **while** $\varepsilon >\bar{\varepsilon }$ **do**

  Mark all *x* ∈ Ω_*n*_ as unassigned

  Set *u*_*i*_ = 0 for *i* ∈ [*n* + 1]

  **while** *some x is marked as unassigned each unassigned* **do**

   **for** *each unassigned x* ∈ Ω_*n*_ **do**

    Calculate i* ∈ arg max_*i*∈[*n*+1]_ Φ_*i*_(*x*) − λ_*i*_

    Calculate j* ∈ arg max_*j*≠i*_ Φ_*j*_(*x*) − λ_*j*_ and set

$$\begin{array}{c} b\left(x\right)=\lambda_{i^{*}}+\varepsilon +\left(\Phi_{i^{*}}\left(x\right)-\lambda_{i^{*}}\right)-\left(\Phi_{j^{*}}\left(x\right)-\lambda_{j^{*}}\right) \end{array}$$

    **if** ∑_*x*_ *u*_i*_(*x*) = *V*_i*_ **then**

     Find $y=\arg\min_{z\in u_{i^{*}}^{-1}\left(1\right)}b\left(z\right)$

     Set *u*_i*_(*y*) = 0 and set *u*_i*_(*x*) = 1

     Mark *y* as unassigned and mark *x* as assigned

     Set $\lambda_{i^{*}}=\min_{z\in u_{i^{*}}^{-1}\left(1\right)}b\left(z\right)$

    **else**

     Set *u*_i*_(*x*) = 1

     **if** ∑_*x*_ *u*_i*_(*x*) = *V*_i*_ **then**

      Set $\lambda_{i^{*}}=\min_{z\in u_{i^{*}}^{-1}\left(1\right)}b\left(z\right)$

  Set *ε* = *ε*/*α*

  **if** $\varepsilon <\bar{\varepsilon }$ **then**

   Set *u*^*s*+1^ = *u*

 Set *s* = *s* + 1

## Two-dimensional numerical examples

### Time-evolution of foams

For an equal-areal, *n* = 12-foam, we show the time evolution corresponding to the gradient flow of the total energy with a random initialization. Here and in subsequent experiments, we generate the random initialization with volume constraints as follows:

1. Generate a random *n*-Voronoi tessellation in a smaller box contained in the whole computational domain and set the complement as *n* + 1-th Voronoi domain.
2. Set $u_{i}=1/{\tilde{V}}_{i}$, *i* ∈ [*N* + 1] where ${\tilde{V}}_{i}$ is the volume of the *i*-th Voronoi domain.
3. Run Algorithm 1 once to get an *n* + 1–partition in the computational domain and set the corresponding indicator functions as the random initial condition.

**Fig 1:**
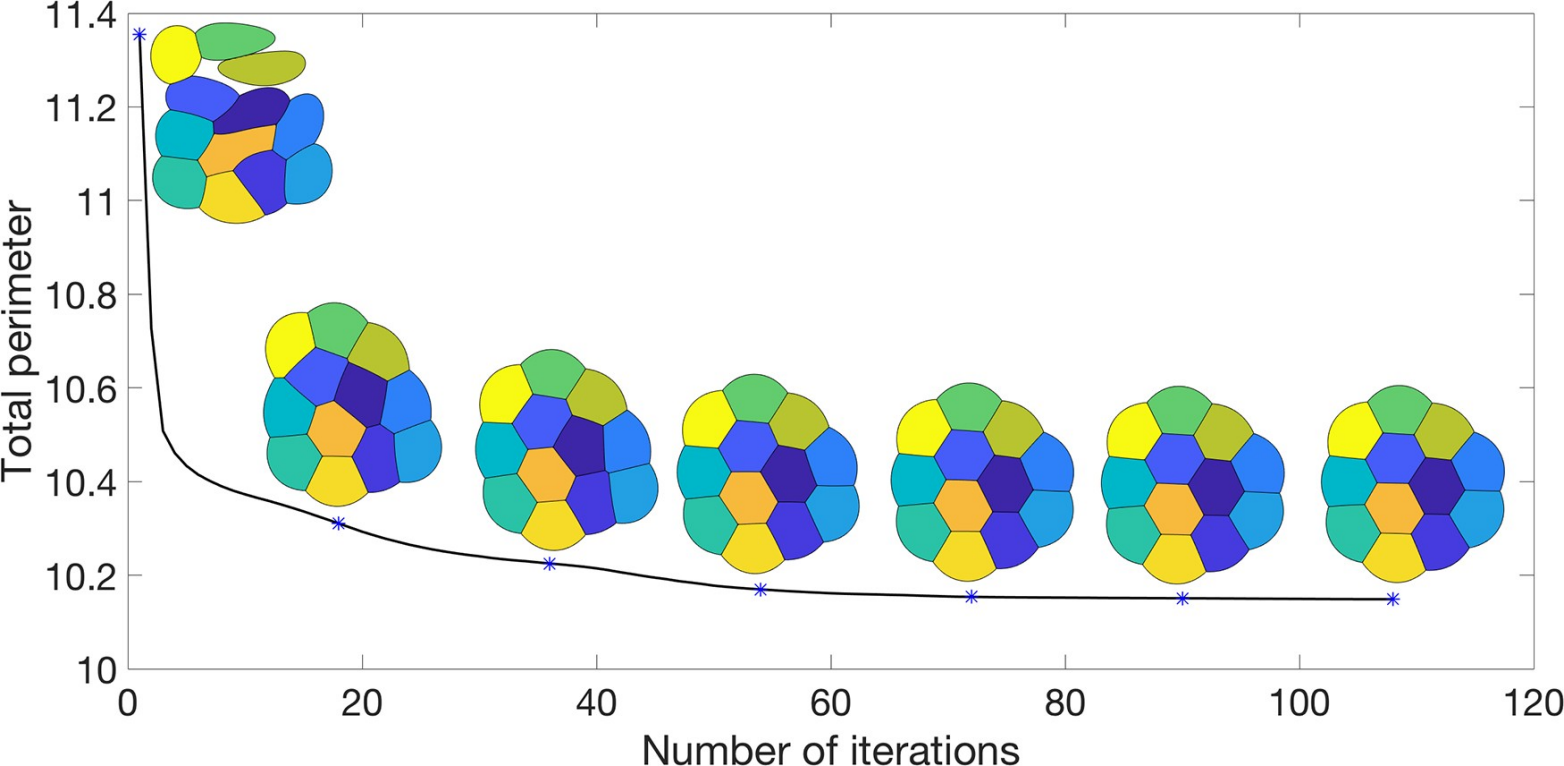
A plot of the energy as a *n* = 12-foam evolves from a random initialization together with the foam configuration at various iterations.

The energy at each iteration is plotted in Fig 1 with the foam configuration at various iterations. Note that the energy decays very fast; in 108 iterations, the configuration is stationary in the sense that no grid points are changing bubble membership. After ≈ 50 iterations, the foam configuration changes very little.

### Stationary solutions

**Fig 2:**
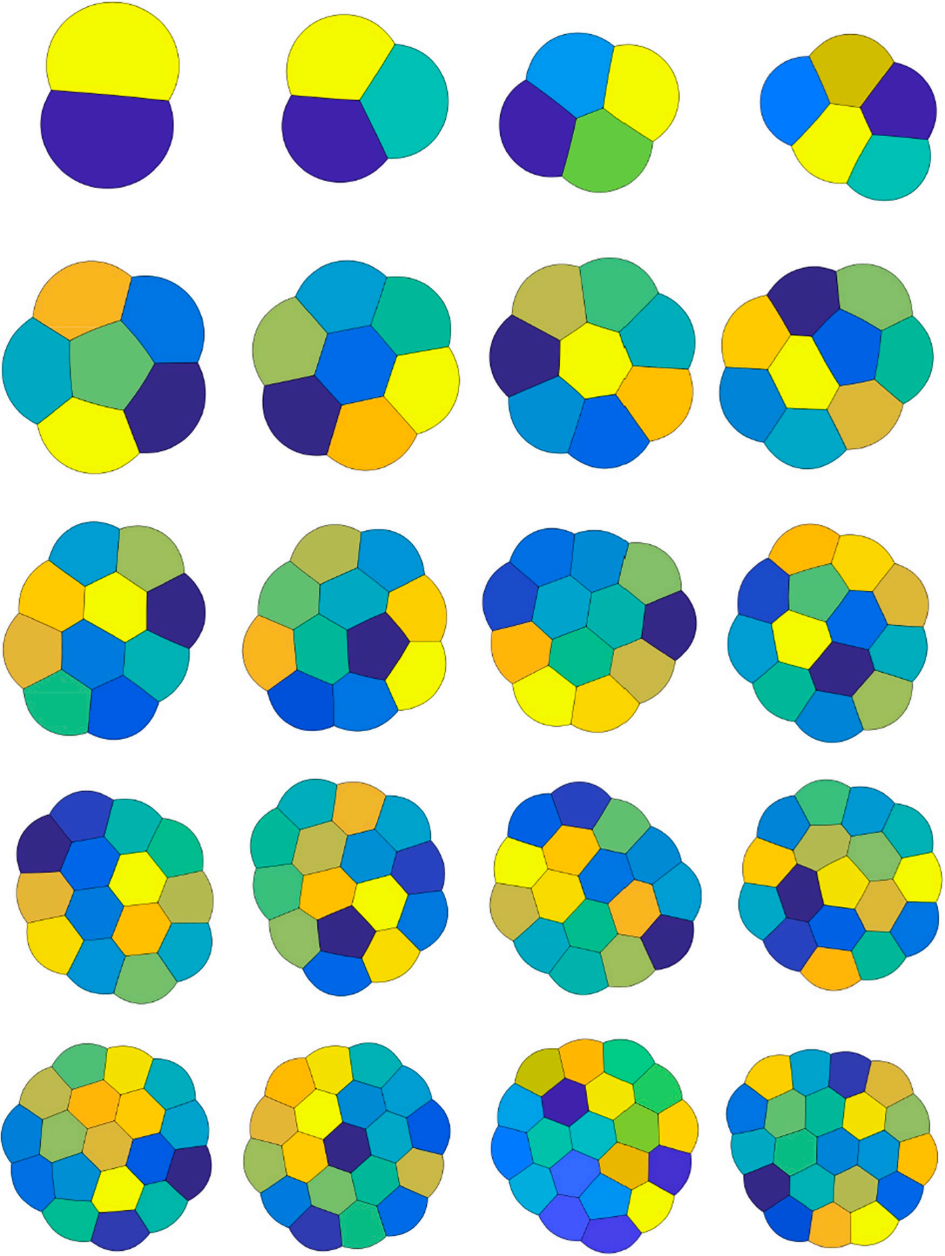
Stationary equal-area *n*-foams for *n* = 2, …21 with smallest computed total perimeter.

**Fig 3:**
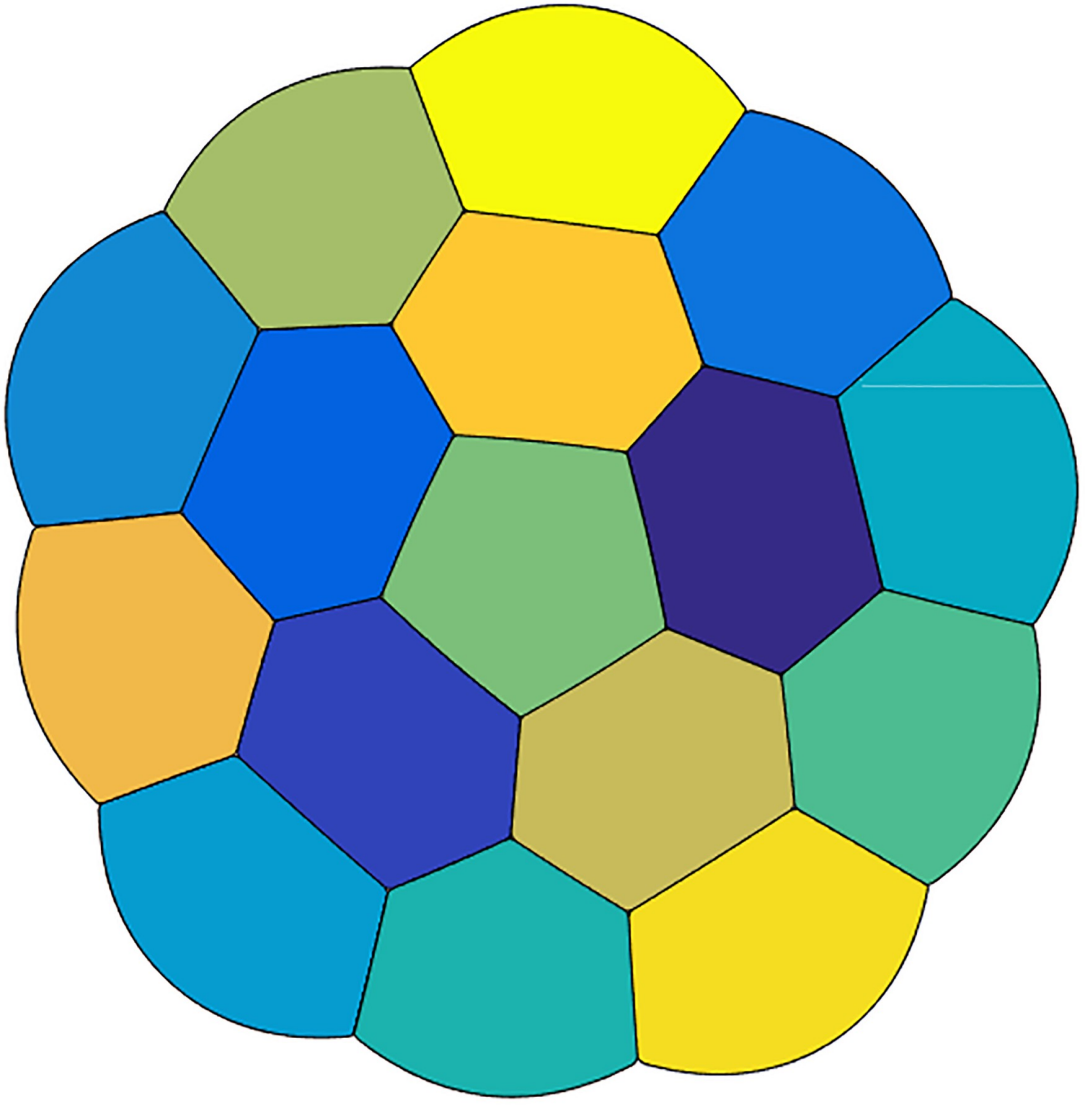
Another stationary equal-area 16-foam with larger total perimeter than the 16-foam displayed in Fig (2).

We consider two-dimensional equal-area foams and evolve many random initial configurations until stationarity. The random initial configurations are chosen as described above. In Fig 2, we plot the *n*-foams with the smallest total perimeter obtained for *n* = 2, …, 21. These results reproduce the results in [3]. We make the following observations:

1. In all cases, Plateau’s necessary conditions for optimality are satisfied.
2. For *n* = 2 and *n* = 3, we obtain the expected double and triple-bubble configurations.
3. For *n*-foams with *n* ≤ 5, there are no interior bubbles and for *n*-foams with *n* ≥ 6, there appears to be at least one interior bubble.
4. For *n* = 6, 7, 8, we obtain *n*-foams with one interior bubble and *n* − 1 boundary bubbles. For *n* = 6 and *n* = 8, due to the 120° angle condition, the interior bubble is not a polygon, but has curved boundary.
5. The configurations for some values of *n* exhibit more symmetry than others. For example, *n* = 10, 16, and 20 display additional symmetries.
6. In Fig 3, another stationary equal-area 16-foam is given with slightly larger (numerically computed) total perimeter than the 16-foam given in Fig 2. It also satisfies Plateau’s laws. Interestingly, the 16-foam in Fig 3 has more rotational symmetries than the 16-foam in Fig 2. It is also more similar to the 17-foam in Fig 2.

### Quasi-stationary flows corresponding to changing bubble size

**Fig 4:**
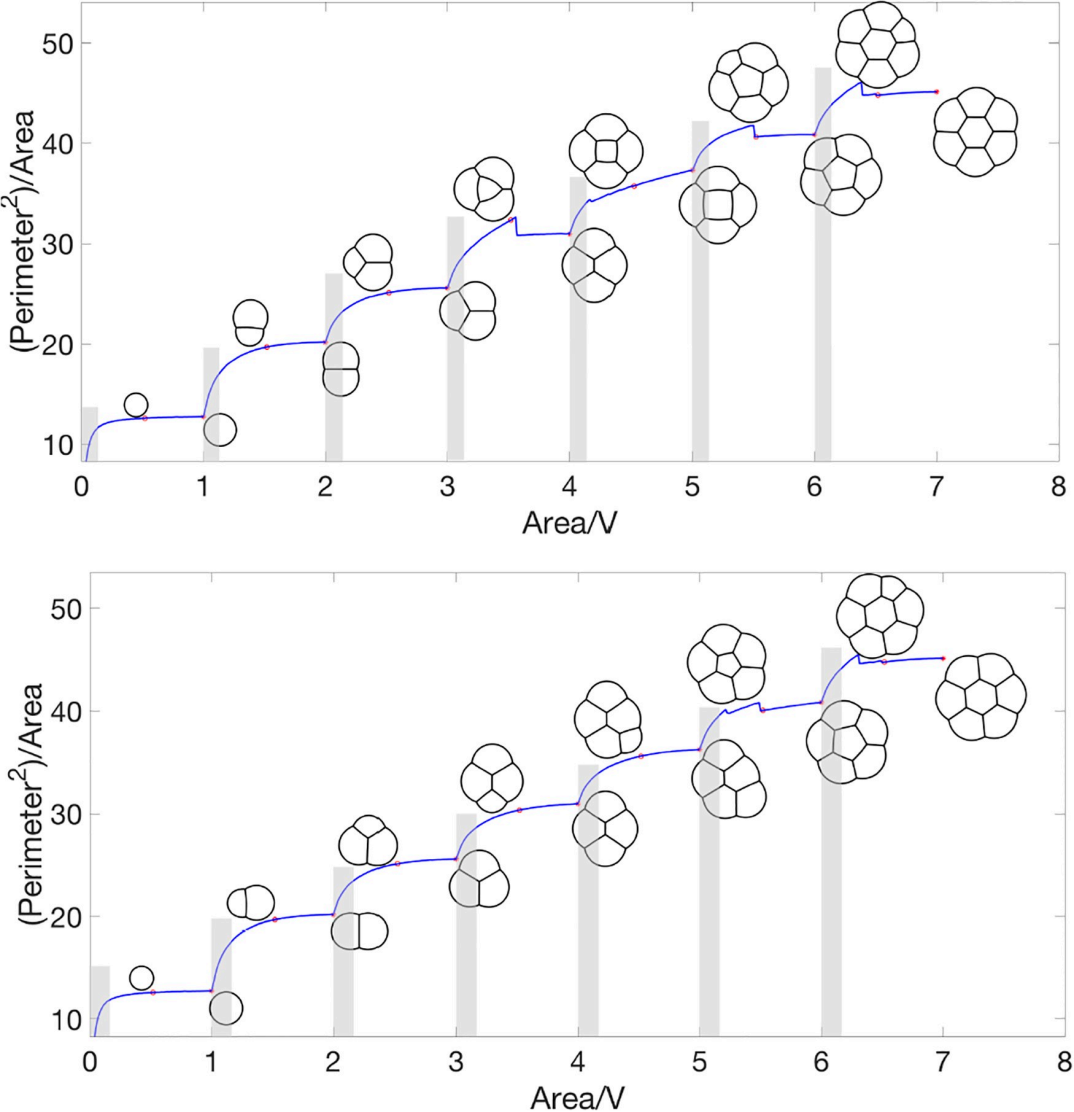
Total perimeter for the quasi-stationary flow, where the area of one of the bubbles is slowly varied and for each fixed area, the stationary solution is computed. When the area reaches *V*, a new bubble with area *v* is introduced. The top and bottom panels correspond to different positions where the new bubble is introduced. The foam configuration at various values of total area is plotted. Links to videos for this quasi-stationary flow are given in Table 1.

We consider the configuration transition by increasing volume by *dV* from only one bubble with small volume (*v*) to a fixed *V* gradually. Then, we add another small bubble on the boundary of the cluster and increase the volume of this small bubble to *V* gradually. By adding bubbles at different positions, we obtain different configuration transitions. Two example quasi-stationary flows are displayed in Fig 4. In this example, *V* = 0.4, *dV* = 0.004, and *v* = 0.016. Links to corresponding videos are given in Table 1.

**Table 1:** 

Quasi-stationary flows corresponding to decreasing the area of one bubble.	
Evolution from a 3-foam to a 2-foam:	youtu.be/LcX9iVE3cEk
Evolution from a 4-foam to a 3-foam:	youtu.be/t44JBQ4Cv9E
Evolution from a 5-foam to a 4-foam:	youtu.be/uyRvH9CpQCM
Evolution from a 6-foam to a 5-foam:	youtu.be/Fs8XF6aNjEg
Evolution from a 7-foam to a 6-foam:	youtu.be/w7p6E2Vcspg
Evolution from a 8-foam to a 7-foam:	youtu.be/s0XNdaJP364
Evolution from a 9-foam to a 8-foam:	youtu.be/XBiQRvjgDVQ
Quasi-stationary flows corresponding to increasing the area of one bubble.	
Evolution from a 2-foam to a 3-foam:	youtu.be/dfPmFPD4Atw
Evolution from a 3-foam to a 4-foam:	youtu.be/cFHXdMwFo7M
Evolution from a 4-foam to a 5-foam:	youtu.be/j7-5L9ff_xg
Evolution from a 5-foam to a 6-foam:	youtu.be/m85uyeiQ2BM
	youtu.be/0KpHnPKl0tA
	youtu.be/jatMSRAxYfQ
Evolution from a 6-foam to a 7-foam:	youtu.be/BP0z93JULCE

Links to videos showing the quasi-stationary flow as the area of one bubble is either increased or decreased. Example foam configurations from this flow are shown in Fig 4.

*Remark* 0.4. The approximation $H^{d-1}\left(\partial \Omega_{i}\cap \partial \Omega_{j}\right)\approx L\left(u_{i},u_{j}\right)$, where *L*(*u*_*i*_, *u*_*j*_) is defined in (6), has *O*(*τ*) accuracy. When the volume of one bubble is *o*(*τ*), this approximation is not very accurate. Of course, to resolve a smaller volume, the accuracy could be improved by using a smaller value of *τ*. However, for a smaller *τ*, the mesh must also be refined to avoid freezing at some non-stationary configuration, which makes the overall algorithm more computationally expensive. In Fig 4, we use gray rectangular boxes to indicate the regime where the results of the algorithm are not very convincing for the value *τ* = 0.0625 used. For example, when there is only one bubble, the isoperimetric quantity, $\frac{{\text{Perimeter}}^{2}}{\text{Area}}$, should be constant (= 4*π*) and our numerical result agrees well with this value outside of the gray region.

### Configuration transitions

The problem of finding minimal total perimeter foams (1) possesses several local solutions corresponding to distinct foam configurations which are well-separated and have almost the same total perimeter. When the problem is perturbed (*e.g*., the volume of one of the bubbles increases or decreases), these local minima vary. As we perturb the problem, we observe *configuration transitions* where a local minima rapidly transitions and converges to another local minima. This is demonstrated in Fig 4, where there are small jumps in the energy curve. In this section we further study this phenomena.

**Fig 5:**
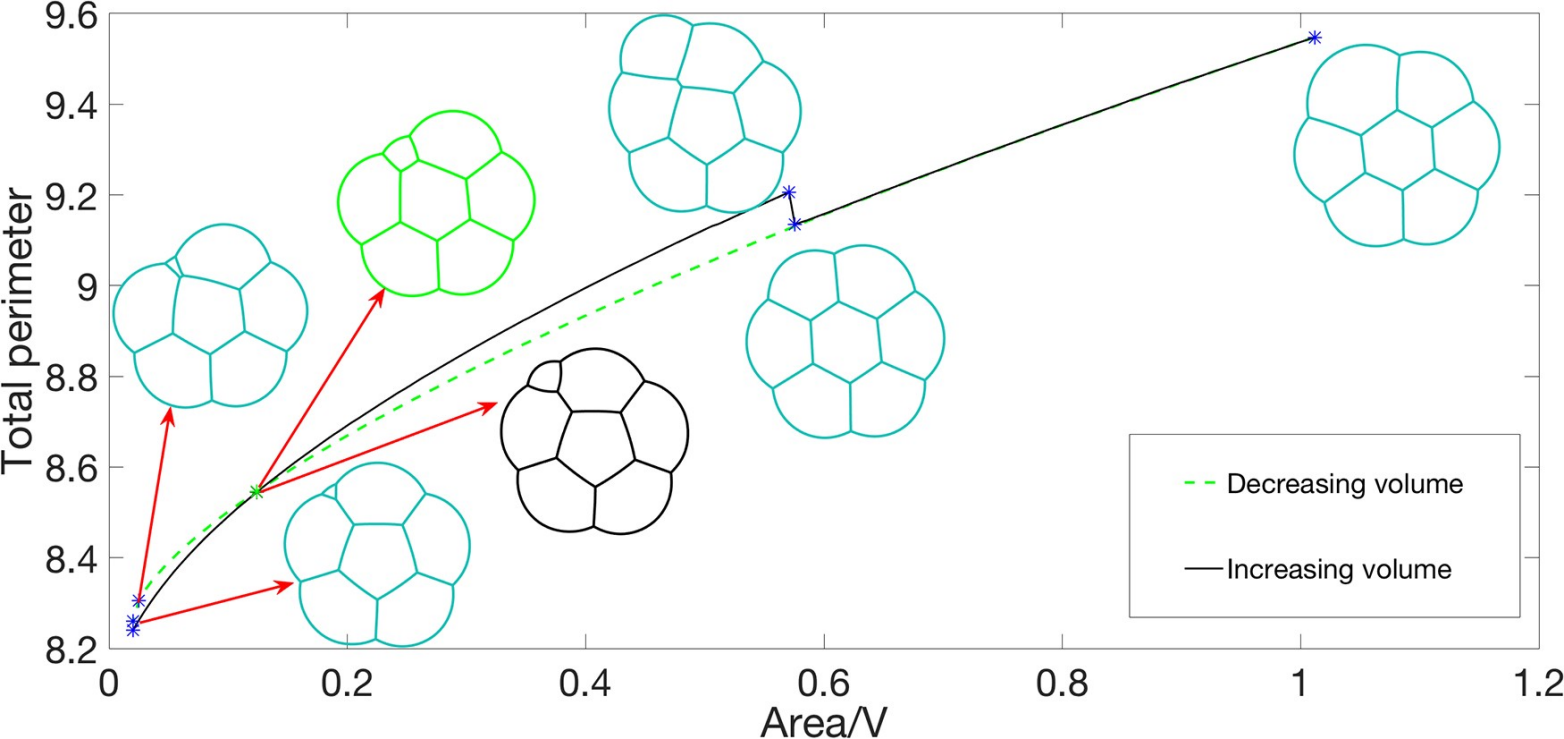
Energy plot of increasing and decreasing area with snapshots at different value of area.

Considering a system with 6 bubbles with equal areas *V* and one small bubble with area *v*, we gradually increase the volume of the small bubble to 1.5*V* and then decrease the volume of this bubble to the original area *v*. In Fig 5, we plot the resulting energy plot and selected configurations in an experiment where *V* = 0.677, *dV* = 0.00496, and *v* = 0.0201. The black line corresponds to increasing area and the green dashed line corresponds to decreasing area. The jumps in the black and dashed green lines are positions of configuration transitions. We also note that the intersection between the black and dashed green lines corresponds to two different configurations, as indicated. These two configurations have the same energy and same bubbles areas. Interestingly, from this experiment, we see that the process of increasing and decreasing volume is *irreversible*; one can view this as a type of *hysteresis* in the sense that the quasi-stationary flow depends on the initialization. That is, as the volume changes, the transition between families of local minima depends on whether the volume is increasing or decreasing.

**Fig 6:**
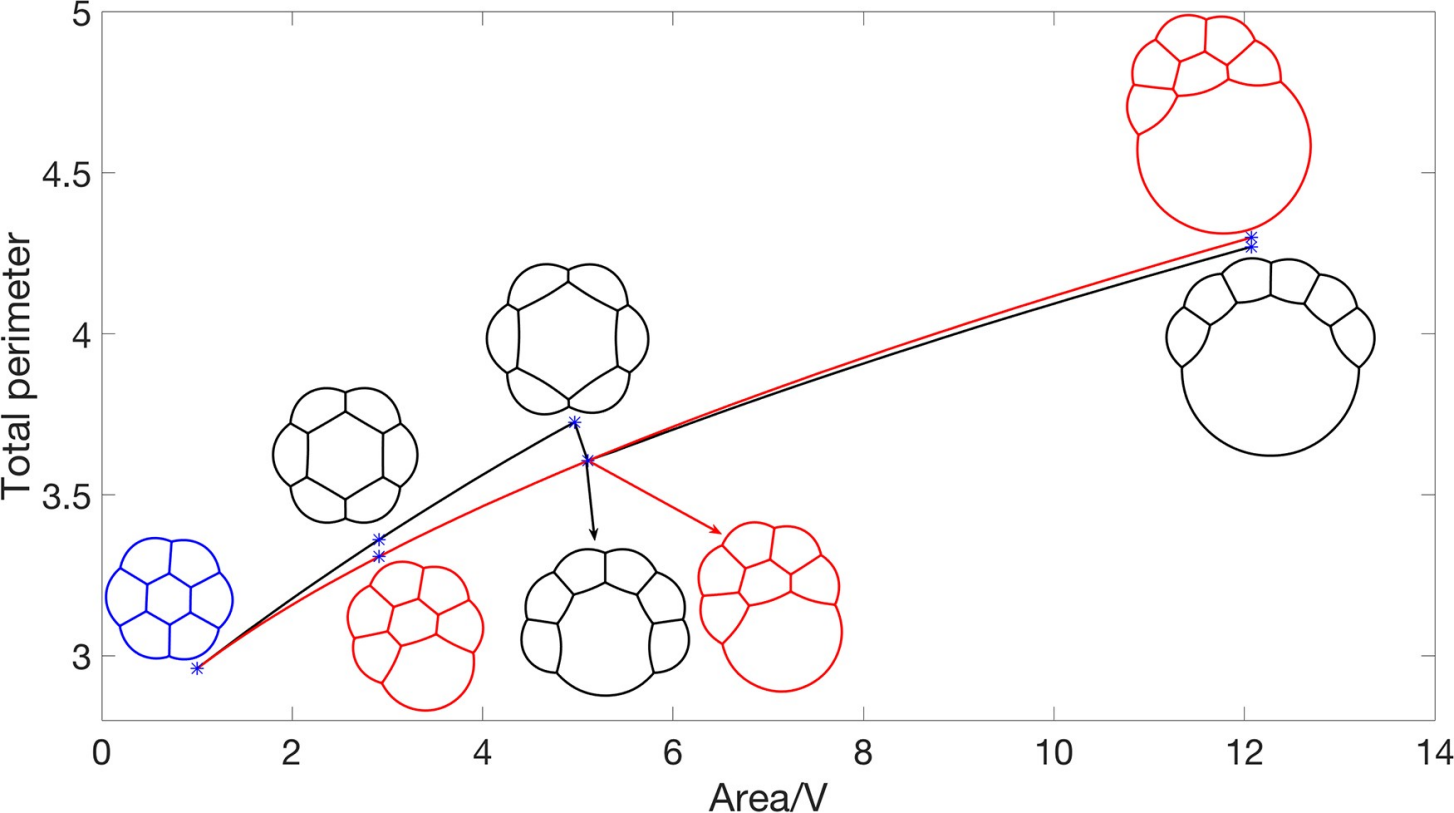
Energy plot of increasing area at different positions with snapshots at different value of area. See S1 Table for links to videos showing the quasi-stationary flow as the area of one bubble is increased either in the middle or the border.

Also, in Fig 4, we observe different stationary configurations when we add area to one bubble at different positions. To further study this, starting from the computed stationary configuration for an equal-area 7-foam (see Fig 2), *V*, we gradually add area to one bubble until the area is 12*V*. We compare the difference between adding area to the middle bubble and adding the area to the border bubble. In Fig 6, the black line displays the change in total perimeter when we increase the area of the middle bubble while the red line displays the change in total perimeter when we increase the area of a border bubble. Snapshots of the configuration when increasing the area of the middle bubble are plotted in black and snapshots of the configuration when increasing the area of a border bubble are plotted in red. In this example, *V* = 0.1474 and *dV* = 0.02. The links for the corresponding videos are also given in Fig 6.

## Three-dimensional numerical examples

### Time-evolution of foams

**Fig 7:**
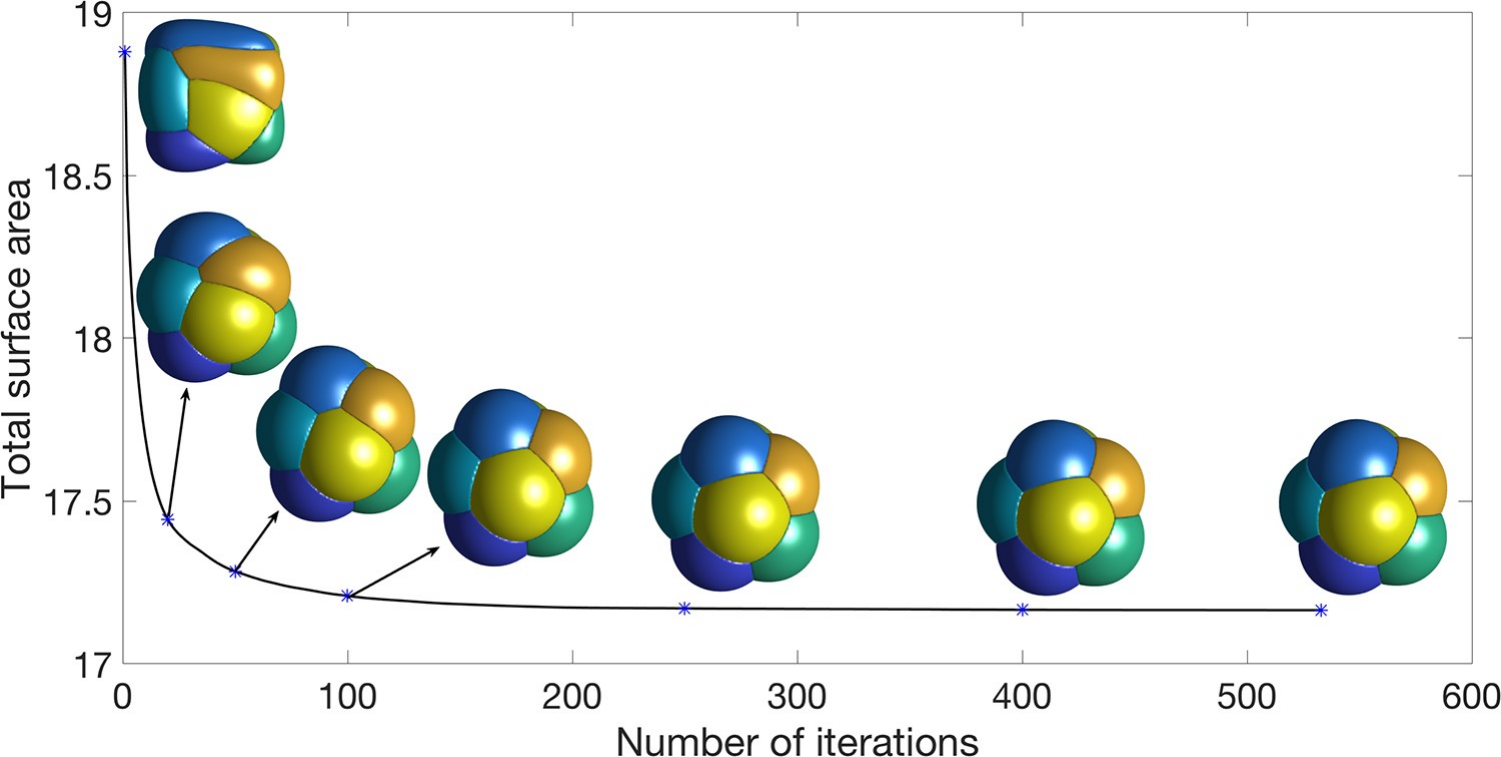
A plot of the energy as a *n* = 8-foam evolves from a random initialization together with the foam configuration at various iterations.

In Fig 7, for an equal-volume, *n* = 8-foam, we show the time evolution corresponding to the gradient flow of the total surface area with a random initialization; the initial configuration was chosen as in the two-dimensional flow. The energy at each iteration is plotted together with the foam configuration at various iterations. Note that the energy decays very fast; even in three-dimensional space, after 533 iterations, the configuration is stationary in the sense that no grid points are changing bubble membership. After ≈ 150 iterations, the foam configuration changes very little.

### Stationary solutions

**Fig 8:**
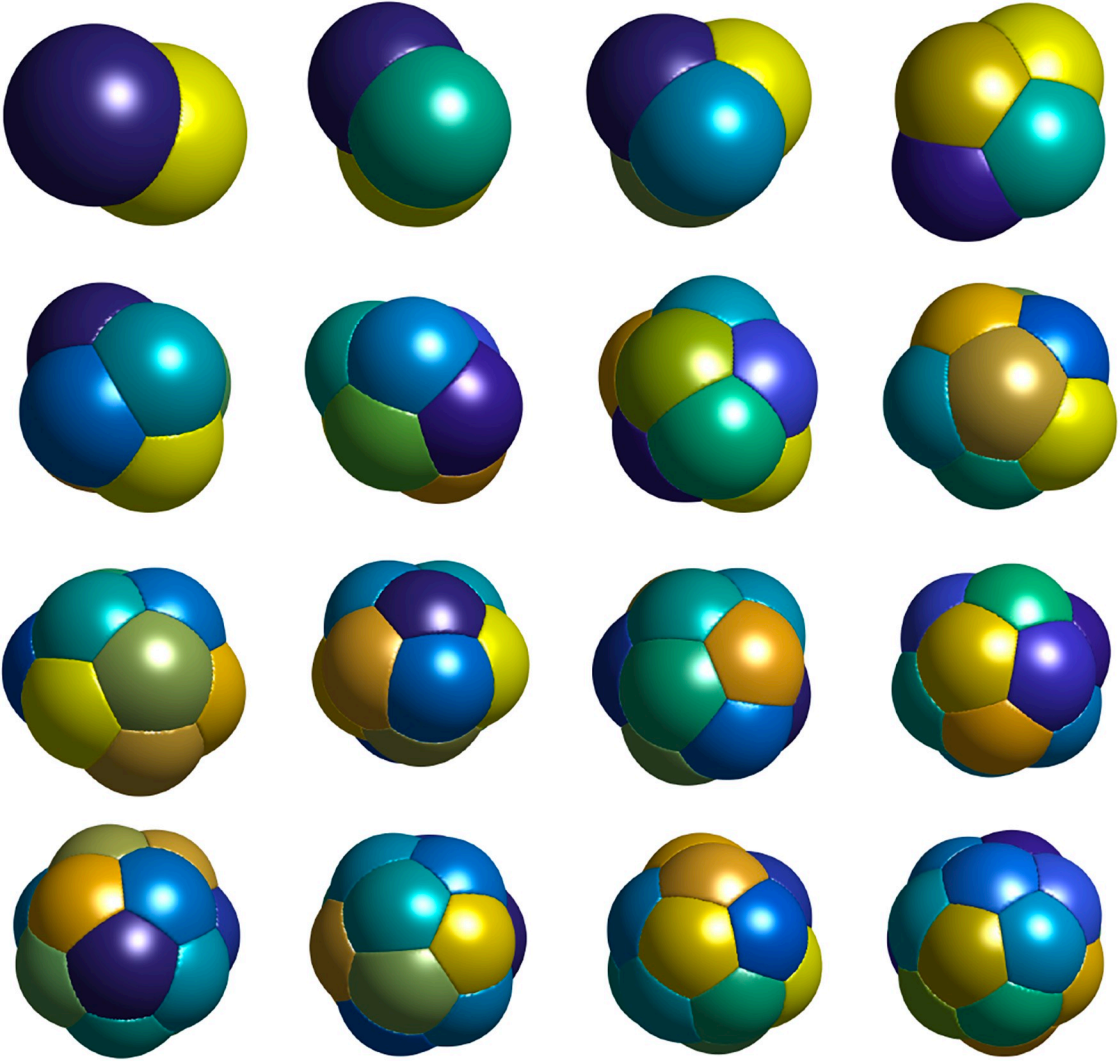
Stationary equal-volume *n*-foams for *n* = 2, …, 17 with smallest computed total surface area. See S2 Table for links to videos illustrating the foam structure.

In Fig 8, we plot the three-dimensional *n*-foams with smallest total surface area found for *n* = 2, …, 17. We make the following observations.

**Fig 9:**
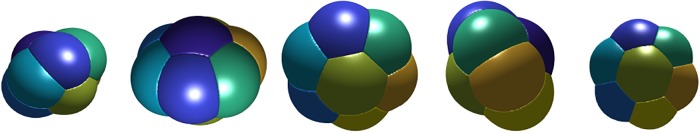
The left panel shows another stationary equal-area 8-foam with larger total surface area than the 8-foam in Fig 8. The middle three panels show *xy*-, *xz*-, and *yz*-views of the 8-foam. The right panel shows another view showing the hexagonal shaped bubble on the top. A corresponding video can be found here: youtu.be/4_uAeq19qJY.

**Fig 10:**
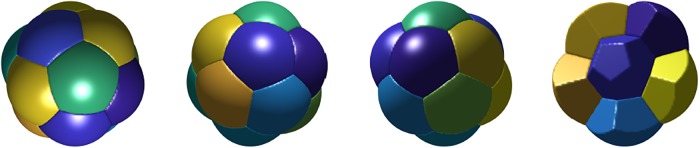
The first three panels show *xy*-, *xz*-, and *yz*-views of the 13-foam in Fig 8. The right panel shows a dissection of this foam, exposing the interior bubble, which is a regular dodecahedron.

**Fig 11:**
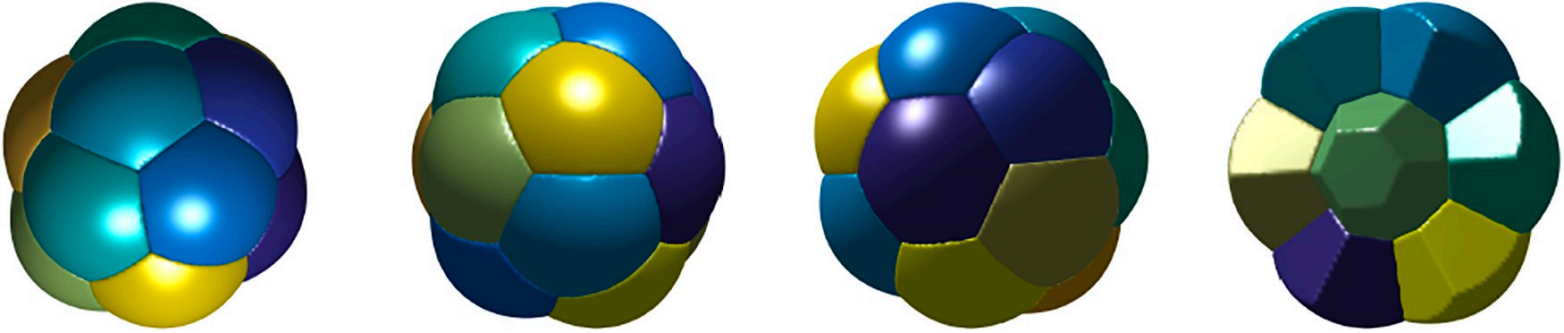
The first three panels show *xy*-, *xz*-, and *yz*-views of the 15-foam in Fig 8. The right panel shows a dissection of this foam, exposing the interior bubble, which is similar to the Weaire–Phelan structure.

1. In all cases, Plateau’s necessary conditions for optimality are satisfied.
2. For *n* = 2 and *n* = 3, we obtain the expected double and triple-bubble configurations.
3. For *n* = 4, the centers of the bubbles form a tetrahedron.
4. For *n* = 5, 6, 7, the *n*-foams consist of two vertically-stacked bubbles with *n* − 2 bubbles arranged with centers in a regular polygon.
5. For *n* = 8, we repeated the experiment with random initial conditions 100 times. In 99 of the experiments, we obtained the 8-foam as shown in Fig 8. In one of the 100 experiments, we obtained another candidate foam which consists of two vertically-stacked bubbles with 6 bubbles arranged with centers in a regular hexagon as shown in Fig 9. The computed total surface area of the configuration in Fig 9 is ≈ 3.8% higher than the stationary 8-foam in Fig 8. This foam also satisfies Plateau’s laws. It is interesting that the algorithm converges to this local minimizer so infrequently, so the basin of attraction for this local minimum is small.
6. For *n*-foams with *n* ≤ 11, there are no interior bubbles and for *n*-foams with *n* ≥ 12, there appears to be at least one interior bubble.
7. The stationary 13-foam is very regular and composed of one interior bubble and 12 bubbles that are on the boundary. In Fig 10, we plot *xy*-, *xz*-, and *yz*-views of the 13-foam and a partial plot of the foam showing the interior bubble. Interestingly, the interior bubble is very similar to a regular dodecahedron. We note that, in a regular dodecahedron, the angle between each two faces is ≈ 117°; we expect the surface of the interior bubble to be slightly curved (non-flat).
8. The 15-foam candidate is also very regular and is composed of one interior bubble and 14 bubbles on the boundary. In Fig 11, we plot *xy*-, *xz*-, and *yz*-views of the 15-foam and a partial plot of the foam showing the interior bubble. The interior bubble is very similar to the truncated hexagonal trapezohedron that appears in the Weaire–Phelan structure. The bubbles on the boundary consist of twelve rounded irregular dodecahedron and two rounded truncated hexagonal trapezohedron.

## Discussion

In this paper, we considered the variational foam model (1), where the goal is to minimize the total surface area of a collection of bubbles subject to the constraint that the volume of each bubble is prescribed. Sharp interface methods together with an approximation of the interfacial surface area using heat diffusion leads to (9), which can be efficiently solved using the auction dynamics method developed in [27]. This computational method was then used to simulate time dynamics of foams in two- and three-dimensions; compute stationary states of foams in two- and three-dimensions; and study configurational transitions in the quasi-stationary flow where the volume of one of the bubbles is varied and, for each volume, the stationary state is computed. The results from these numerical experiments are described and accompanied by many figures and videos.

The methods considered in this paper could be used to simulate foams where the bubbles have different surface tensions or different surface mobilities using the modifications developed in [28].

In Remark 0.4, we observed that for small bubbles, a small time step *τ* must be used and consequently a fine mesh. Also, the computational cost for this algorithm increases with the number of bubbles. Finding ways to extend this method to small bubbles and large number of bubbles is challenging and beyond the scope of this paper.

One question that we find intriguing is: for fixed k∈N, how many bubbles in an equal-area stationary foam are needed before there are *k* in the interior? In two-dimensions, we observe that 6 bubbles are needed for one interior bubble, 9 are needed for two, 11 are needed for three, etc…. In three-dimensions, 12 bubbles are needed for one interior bubble. Numerical evidence suggests that more than 20 bubbles are needed before two interior bubbles appear.

We hope that the numerical experiments conducted in this paper and further experiments using the methods developed can provide insights for further rigorous geometric results for this foam model.

## Supporting information

S1 Table (PDF)

S2 Table (PDF)
