# Supplementary material for: Dynamics and stationary configurations of heterogeneous foams
Source: PLoS One. 2019 Apr 29;14(4):e0215836. doi: 10.1371/journal.pone.0215836 (PMC6488059; doi:10.1371/journal.pone.0215836)
Supplement: S1 Table — (PDF) [file pone.0215836.s001.pdf]

| | |
|---|---|
| Increasing the area of the middle bubble: | youtu.be/–HWXssRERk |
| Increasing the area of a border bubble: | youtu.be/cJsbU1mtT3E |
