# Supplementary material for: Dynamics and stationary configurations of heterogeneous foams
Source: PLoS One. 2019 Apr 29;14(4):e0215836. doi: 10.1371/journal.pone.0215836 (PMC6488059; doi:10.1371/journal.pone.0215836)
Supplement: S2 Table — (PDF) [file pone.0215836.s002.pdf]

2-foam:	youtu.be/UrqE4vmkxnc	3-foam:	youtu.be/oQxR6Z_fpTE
4-foam:	youtu.be/LQEphY2Ctq4	5-foam:	youtu.be/EDEwdMR21Xo
6-foam:	youtu.be/-PlS75F6Ueo	7-foam:	youtu.be/Avke4wfKADY
8-foam:	youtu.be/tI1zb685MAs	9-foam:	youtu.be/eu2OYEC7KUE
10-foam:	youtu.be/J9mlXTLNuxc	11-foam:	youtu.be/pMCCIQzVEqk
12-foam:	youtu.be/-cHEncU2a7o	13-foam:	youtu.be/bMBFzjJ-3wY
14-foam:	youtu.be/Rj9VfPd9Trc	15-foam:	youtu.be/atUOXP0FtcA
16-foam:	youtu.be/QZRtyG-fOb0	17-foam:	youtu.be/AHjbckdh5EY
